# Identification and Validation of Quantitative PCR Reference Genes Suitable for Normalizing Expression in Normal and Dystrophic Cell Culture Models of Myogenesis

**DOI:** 10.1371/currents.md.faafdde4bea8df4aa7d06cd5553119a6

**Published:** 2014-03-06

**Authors:** John C.W. Hildyard, Dominic J. Wells

**Affiliations:** Department of Comparative and Biomedical Sciences, Royal Veterinary College, London, UK; Department of Comparative and Biomedical Sciences, Royal Veterinary College, London, UK

## Abstract

The coordinated differentiation of myoblasts to mature muscle is essential for muscle development and repair, and study of the myogenic program in health and disease is critical to the understanding and treatment of muscle pathologies. Use of quantitative RT-PCR to analyse gene expression in cell culture models of muscle differentiation can be highly informative, but data must be normalized to one or more suitable reference genes. Myogenesis is highly dynamic, thus identification of genes with stable expression throughout this process is challenging. Establishing a common set of reference genes suitable for measuring expression in both healthy and disease models would be of considerable advantage. We measured expression of 11 candidate normalization genes (Cdc40, Htatsf1, Ap3d1, Csnk2a2, Fbxw2, Fbxo38, Pak1ip1, Zfp91, GAPDH, ActB, 18S) in three cell culture models of myogenesis (C2C12 , H2K2B4, and the dystrophic line H2KSF1). Strong and weak normalization candidates were identified using the software packages Bestkeeper, geNorm and Normfinder, then validated against several known myogenic markers (MyoD, myogenin, MEF2C, dystrophin). Our data show that Csnk2a2 and Ap3d1 are suitable for normalizing gene expression during differentiation in both healthy and dystrophic cell-culture models, and that the commonly-used reference standards 18S, ActB and GAPDH are exceptionally poor candidates.

## Introduction

Quantitative PCR (qPCR) is a powerful tool for measuring changes in gene expression quickly and affordably, exploiting the exponential nature of PCR to obtain straightforward numerical data from cDNA prepared using even very small amounts of starting mRNA. However the effects of inherent minor variations in sample preparation and handling, and variable efficiencies of RNA purification and cDNA synthesis^1^, can combine to produce significant alterations in measured values. To meaningfully interpret quantitative PCR data for a given gene of interest the use of internal reference genes is therefore essential. Highly-expressed “housekeeping” markers such as beta-actin (**ActB**), **GAPDH** or **18S** ribosomal RNA are widely employed, with the assumption that these markers remain constant for any given mRNA population within an experiment. There is growing evidence to suggest, however, that even these established markers may vary significantly under many experimental conditions, particularly those involving global changes in cell behaviour^2^^,^ 3 ^,^ 4 . Actin and GAPDH commonly exhibit strong stability at the protein level, but this stability is not necessarily a useful indicator of stability at the mRNA level, where turnover is inherently more dynamic. Use of ribosomal RNA or other non-coding RNApolI/III transcripts carries further caveats, as these RNAs are often subject to different pathways of synthesis and degradation^5^^,^ 6, frequently exhibit extensive secondary structure (potentially impairing cDNA synthesis), are intrinsically linked to the rate of cellular activity, and -of critical relevance- comprise a disproportionately large fraction of total RNA (approx 80-95% depending on conditions^7^). As a consequence, changes in levels of these RNAs may mask or even exaggerate changes at the mRNA level, and thus are potentially poor choices for normalization.

To obtain quantitative PCR data of high confidence, it is thus sensible to assume there is no true “one size fits all” reference gene, and instead determine which genes exhibit the greatest stability for the experimental conditions in question. Use of multiple reference genes, preferably associated with independent cellular processes, is recommended: this increases confidence that any observed differences are accurate reflections of total mRNA levels rather than gene-specific perturbations due to experimental conditions.

**Gene expression during myogenesis**

Cell culture models of myogenesis are popular tools for the study of muscle development, repair and response to physiological and pharmacological agents. Progress though the myogenic program from proliferating myoblasts to terminally-differentiated myotubes necessitates major shifts in morphology and cellular activity: a complete cessation of the cell cycle, followed by cell migration and fusion into multinucleated myotubes (Fig 1). These subsequently mature through further fusion events, progressively lengthening, thickening and producing large quantities of contractile proteins, and ultimately are capable of developing spontaneous contractility. This process is likely to produce wide-ranging changes in gene expression, including those of metabolic and cytoskeletal components, members of which (**GAPDH** and beta-actin, respectively) are frequently selected as normalization genes. Selection of an appropriate set of reference genes under such a varied suite of conditions is thus non-trivial, yet nevertheless critical for accurate measurement of gene expression during myogenesis. A number of studies have identified candidate reference genes for use in studies of mature muscle (and in cultured, terminally-differentiated myotubes) in a range of species^8^^,^ 9 ^,^ 10 ^,^ 11 ^,^ 12 , though candidates for normalization of gene expression throughout the myogenic program (particularly in cell culture) are more limited^13^^,^ 14, and are generally specific to a single cell line. Furthermore, such studies typically use at most 5 or 6 candidate genes (occasionally as few as 3), and do not necessarily validate the candidates identified against multiple genes with known expression patterns.

**Progression through myogenesis:**
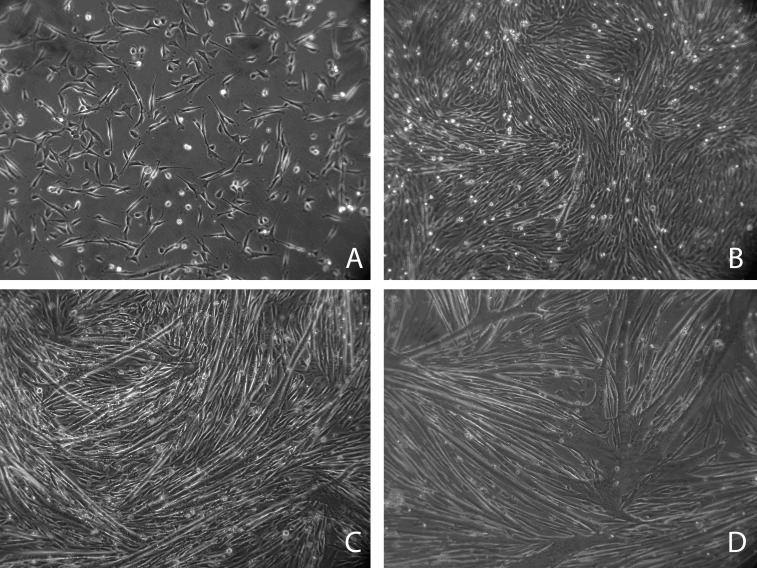
H2KSF1 myoblasts in proliferation medium (A), and 24, 48 and 72 hours after initiation of differentiation (B-D, respectively). Mononucleated myoblasts (A) fuse to form multinucleated myotubes of increasing size (BCD).

We examined gene expression of a comparatively large set of putative reference genes throughout myogenic differentiation in two healthy and one disease-model murine cell culture systems: the commonly-used **C2C12** myoblast line^15^ , and the immortomouse-derived myoblast lines **H^2K^2B4** 16 and **H^2K^SF1** 17 . The latter cell line (**SF1**) carries the *mdx* mutation (a premature termination codon in exon 23 of the dystrophin gene) and is thus a cell line model for dystrophic muscle.

We here present data suggesting a common set of candidates for normalizing gene expression during myogenesis in these three culture models of murine muscle differentiation.

For reference gene candidates, eleven genes were selected from the PrimerDesign geNorm^plus^ reference set (**Cdc40**, **Htatsf1**, **Ap3d1**, **Csnk2a2**, **Fbxw2**, **Fbxo38**, **Pak1ip1**, **Zfp91**, **GAPDH**, **ActB**, **18S**: selected based on their high stability in a large number of systems^18^ , Table 1). This set conveniently includes the three commonly used reference genes noted above, allowing direct assessment of their accuracy in a myogenic model. Relative expression values for the candidate genes were prepared and subsequently analysed for suitability using several freely available software packages: Bestkeeper^19^ , geNorm^20^ and Normfinder^21^ .

To test the efficacy of the genes identified, they were used to normalize measured expression levels of several genes known to change in a recognisable fashion throughout myogenesis (MyoD, myogenin, MEF2C and dystrophin).

**Table 1. Reference genes selected:** The full names and functions of the geNorm^Plus^ genes used in this study

Gene Name	Full Name	Function
^**Cdc40**^	Cell Division Cycle 40/PRP17	Catalytic component of the spliceosome complex
^**Htatsf1**^	HIV-1 Tat Specific Factor	Transcription factor, involved in transcriptional elongation
^**Ap3d1**^	Adapter-Related Protein Complex 3 Subunit	Non-clathrin associated subunit of the AP3 adaptor complex
^**Csnk2a2**^	Casein Kinase 2, Alpha Prime Polypeptide	Subunit of Casein Kinase 2, a Ser/Thr kinase involved in multiple cellular processes
^**Fbxw2**^	F-Box And WD-40 Domain-Containing Protein	Substrate-recognition component of the SCF (SKP1-CUL1-F-box protein)-type E3 ubiquitin ligase complex
^**Fbxo38**^	F-Box Protein 38/MOKA	Putative ubiquitin ligase
^**Pak1ip1**^	PAK1 Interacting Protein 1/PIP1/WDR84	Negative regulator of Pak1 kinase
^**Zfp91**^	Zinc Finger Protein 91/PZF	Atypical E3 ubiquitin-protein ligase that mediates activatory 'Lys-63'-linked ubiquitination of MAP3K14/NIK
^**GAPDH**^	Glyceraldehyde 3-phosphate dehydrogenase	Catalyses conversion of glyceraldehyde 3-phosphate to 3-phospho glyceroyl phosphate
^**ActB**^	Beta Actin	Major component of the non-muscle cytoskeleton
^**18S**^	18S ribosomal RNA	RNA component of the 40S ribosome

## Experimental

**Cell culture:**

H^2K^2B4/SF1 cell lines are derived from immortomouse muscle, and are thus conditionally immortal, expressing a temperature-sensitive SV40 largeT antigen under the control of a gamma-interferon (γ-IFN)-inducible promoter. Cells were grown in matrigel-coated flasks (0.1mg.ml^-1^) and maintained in a proliferative state by incubation at 33°C in proliferation medium: DMEM+glutamax (invitrogen) supplemented with 20% (v/v) heat inactivated-foetal bovine serum, 2% (v/v) chicken embryo extract (CEE, Sera laboratories international), 1% (v/v) Penicillin/Streptomycin (Sigma, final concentration 100u.ml^-1^ penicillin, 100ug.ml^-1^ streptomycin), and 20 U/mL γ-IFN (Chemicon). One day prior to differentiation, cells were seeded onto matrigel-coated 6-well plates at 2x10^5^ cells.well^-1^ (2B4) and 5x10^5^ cells.well^-1^ (SF1), plating conditions established to allow optimal differentiation without under- or overcrowding cells. Differentiation was initiated by replacement of growth media with differentiation medium (DMEM+glutamax supplemented with 5% horse serum (PAA) and 1% pen/strep) and incubation at 37°C. Differentiation medium was partially replaced (50% of medium aspirated, replaced with fresh differentiation medium) after 5 days of differentiation.

C2C12 cells were grown in DMEM+glutamax supplemented with 10% bovine serum and 1% pen/strep. Prior to differentiation, cells were seeded onto 6-well plates and allowed to reach 80-90% confluency. Differentiation was initiated by replacement of growth media with differentiation medium (DMEM+glutamax supplemented with 2% horse serum and 1% pen/strep). Differentiation medium was partially replaced (see above) every two days.

Cells were harvested for RNA (see below) at the time of differentiation and at 24-hour intervals following, until spontaneous contractility was observed by microscopy (between 144 and 168hours post-differentiation): contractile behaviour usually results in subsequent detachment from the plate surface, thus this was a necessary endpoint. Three replicate samples were collected per cell line, per time point.

**QPCR Primers:**

Sequences of reference gene primers (Cdc40, Htatsf1, Ap3d1, Csnk2a2, Fbxw2, Fbxo38, Pak1ip1, Zfp91, GAPDH, ActB) are proprietary property of PrimerDesign^18^ , but generate mouse-specific amplicons of 90-180bp. All other sequences are shown below. Primers to MyoD, myogenenin, MEF2C and dystrophin were designed using primer3 software version 0.4.0 (bioinfo.ut.ee/primer3-0.4.0/) to produce mouse-specific amplicons of 100-220bp, spanning one or more exon/exon boundaries, with a preference for those spanning large introns to prevent amplification of trace genomic DNA carryover. 18S primer sequences were taken from Schmittgen *et al* 3 . All primers had melting temperatures (Tm) of 58-61°C. Amplification of a single, specific band per primer pair was confirmed via conventional PCR for all primer pairs used.

Primer sequences: MyoD Fwd: TACAGTGGCGACTCAGATGC, MyoD Rev: GAGATGCGCTCCACTATGCT, myogenin Fwd: CTACAGGCCTTGCTCAGCTC, myogenin Rev: ACGATGGACGTAAGGGAGTG, MEF2C Fwd: GCCGGACAAACTCAGACATT, MEF2C Rev: TGGGATGGTAACTGGCATCT, dystrophin Fwd: GTGGGAAGAAGTAGAGGACTGTT, dystrophin Rev: AGGTCTAGGAGGCGTTTTCC, 18S Fwd: GTAACCCGTTGAACCCCATT, 18S Rev: CCATCCAATCGGTAGTAGCG

**RNA isolation and cDNA synthesis:**

Total RNA was isolated by addition of 100ul TRIzol (Fisher Scientific) to each well of cells, agitation with a cell scraper and transfer to a clean microcentrifuge tube. Phase-separation was initiated by addition of 30ul of chloroform and vortexing followed by centrifugation. Aqueous phases were collected and nuclease-free water added to a final volume of 500ul (for ease of manipulation), before two sequential chloroform extractions (1:1). RNA was precipitated by addition of 10ug glycogen (Roche), 50ul 3M NaAc pH 5.5, and 1.5ml ice-cold ethanol. Following incubation (1 hour, -80°C) RNA was collected by centrifugation, pellets were washed using ice-cold 70% ethanol and dissolved in nuclease-free water. RNA integrity was established by gel electrophoresis and RNA purity by nanodrop spectroscopy. Acceptable samples displayed two clear ribosomal bands (indicating no degradation occured during isolation), 260/280 ratios > 2.0 and 260/230 ratios > 1.8. Samples with 260/230 ratios below 1.8 (chiefly guanidium carryover, not uncommon when dealing with small TRIzol volumes) were diluted in nuclease-free water to a final volume of 500ul, extracted a second time with chloroform (1:1) and ethanol precipitated as before. This additional extraction reliably produced 260/230 ratios > 2.0, albeit with substantially reduced RNA yields. 1ug of total RNA was used for cDNA synthesis using the RTnanoscript kit (PrimerDesign) according to the manufacturers’ instructions, using both oligo dT and random 9mers as first-strand synthesis primers. cDNA preparations were diluted 20-fold prior to quantitative PCR analysis, and the same preparations were used for all studies.

**QPCR:**

QPCR analyses used Brilliant II SYBR green mastermix (Agilent) or Precision SYBR green mastermix (PrimerDesign) in 384 well plates (white hard-shell thin wall, BioRad) with a CFX384 lightcycler (Biorad) using three-step cycling conditions. Reactions were performed in duplicate or triplicate in 10ul volumes using 1-3ul of diluted cDNA (see above: ca. 2-6ng cDNA assuming 1:1 synthesis) per well. Dilution series were performed to establish that residual cDNA synthesis reagents did not impair PCR efficiency at these dilutions. All runs included melt curve analysis and template-free controls to confirm specific, single-product amplification. All primer pairs produced single amplicons and reactions were of comparable efficiency (95-100%) as established by standard dilution curve and analysis of individual traces.

Quantification cycle (Cq) values were determined by regression analysis of the amplification traces. Average Cq values for each replicate reaction were used either directly (BestKeeper) or internally normalized to a relative linear expression range (0-1) before analysis (Normfinder, geNorm). Samples exhibiting more than 10-fold deviation in relative expression from the dataset average in a majority of genes were typically excluded from analysis. Experimental normalizations using suitable candidates used the linearised expression data of the respective genes, or where multiple normalization genes were used, the geometric average of the linearised expression data.

**Bestkeeper** is available as a prepared excel spreadsheet file (obtained at http://www.gene-quantification.de/bestkeeper.html) into which raw Cq data (from reference candidates and/or genes of interest) is entered, and pairwise comparisons and correlation coefficients are calculated automatically. Documentation is available at the download location. **Normfinder** is available as an excel plugin or as code formatted for R (obtained at http://moma.dk/normfinder-software). Documentation is available at the download location.** geNorm** (as first released) is no longer freely available, having been integrated into the qBase software package (http://www.biogazelle.com/qbaseplus) however an office 2010-compatible copy of the original excel macro program is available at http://ulozto.net/xsFueHSA/genorm-v3-zip. All analysis in this manuscript was performed on a Windows 7 operating system using excel 2003 and excel 2010, and the authors cannot confirm Mac-compatibility of any of the above software packages.

## Results and Discussion

Identification of suitable normalization genes presents a circular conundrum: how can one assess the stability of a given reference gene without a suitable reference gene for comparison? The three software packages employed utilise subtly different strategies to identify suitable reference genes:

- Bestkeeper^19^ calculates a geometric average for the entire dataset provided, thus generating a hypothetical global normalization factor, the ‘bestkeeper’: taking the premise that as the number of genes measured increases, variations resulting from genuine biological changes in gene expression make progressively lower contributions to the overall trend than do global variations in cDNA concentration arising from sample preparation/handling. Thus, by making pairwise comparisons between expression data from individual genes and this ‘bestkeeper’ one can identify those with least variation, i.e. those that best reflect overall cDNA (thus mRNA) levels.
- geNorm^20^ takes a similar approach; reasoning that highly-similar patterns of variation observed in genes associated with unrelated cellular processes (such as those supplied in the geNormPlus gene set) is more likely to reflect variation in cDNA levels than in gene expression. This technique thus utilises pairwise comparison to rank candidate genes by summed individual variation, then iteratively discards the most variant gene before repeating the analysis, thus identifying a pair of genes with minimum respective variation, and a ranked list of genes of increasing variance.
- Normfinder^21^ (unlike the two methods above) does not make direct gene-to-gene comparisons, instead calculating individual variations for each gene relative to the dataset average, accounting for both intergroup (here, comparing different timepoints) and intragroup (comparing replicate timepoints) variation, summing these to provide a gene-specific stability value for a given set of conditions. This inclusion of group-specific analysis thus identifies genes that show the greatest overall stability *over the conditions presented*, rather than those that merely exhibit well-matched variation. The analysis provides both the best single candidate, and the best pair of genes (which may not include the best single candidate: two marginally less-stable genes that vary in opposing fashions may average to provide more stable reference data than a single gene alone).

Both Bestkeeper and geNorm place emphasis on pairwise techniques thus can potentially misattribute value to groups of highly-variable genes with closely-matched expression patterns: co-ordinately regulated cell-cycle factors would score highly, despite being clearly variable. Furthermore, gene-to-gene comparison analysis is highly sensitive to the dataset presented: removal of a single high-scoring candidate lowers the correlation of similar high-scoring candidates, resulting in lower-ranked candidates demonstrating higher relative pairwise correlation, thus potentially ‘reshuffling’ the entire stability curve (similarly, inclusion of additional candidates may create new high-scoring pairings, with similar consequences). However, as pairwise analysis is essentially blind to *overall* apparent signal stability, these techniques are inherently tolerant of ‘messy’ datasets resulting from variable mRNA quality or cDNA synthesis.

Conversely, Normfinder avoids direct gene-to-gene comparison, instead focusing on individual gene stability: the analysis compares each gene individually to group-wide averages, and is thus inherently less sensitive to removal or addition of candidates. While calculating separate values of intragroup (replicate samples) and intergroup (treatment-to-treatment) variance allows estimates to be made of sample variability vs gene stability, this technique necessarily makes the assumption that the dataset averages do not exhibit systematic intergroup variation, and thus is potentially less suitable to datasets derived from samples undergoing progressive cellular alterations (such as the myogenic program).

By using all three techniques for this study, we aimed to minimise the limitations of any specific method while capitalising on the unique strengths of each approach. Furthermore (as these techniques benefit progressively from larger datasets) rather than four or five candidates as is common in the literature, eleven were used. This affords both greater confidence in suggested reference genes, and produces a greater range between those identified as good candidates and those as poor candidates. This latter aspect can be helpful to the researcher, as it is sometimes troubling to simply accept the output of a given mathematical algorithm (especially if the algorithm serves only to rank multiple apparently appropriate reference genes in order of preference). Identification of poor candidates that can be empirically demonstrated as such confers greater strength to the identification of good candidates.

**geNorm** analysis (see Fig 2) indicated only slight consensus on *optimal* normalization candidates between the three cell lines. However, as a commonly accepted threshold for average stability (M) is a value < 0.5, the analysis reveals that *all* normalization genes are potentially adequate candidates in C2C12s. Similarly 2B4 and SF1 cells, while exhibiting lower expression stability than C2C12 (i.e. greater M values), still present relatively few candidates failing to fall below the 0.5 threshold. **Pak1ip1**, **Csnk2a2** and **Ap3d1** rank relatively highly in all three cell lines, while other genes vary considerably in their relative stabilities. Of additional note, both **18S** and **ActB** rank very poorly in all lines, while **GAPDH** ranks highly in SF1 cells alone. geNorm additionally calculates the pairwise variation of normalization factors derived from increasing numbers of genes, allowing estimation of the number of control genes needed for optimal normalization: as a pairwise analysis, 2 genes is the minimum. Additional normalization genes may reduce variation, but this is not guaranteed, nor indeed always necessary. Values below 0.15-0.2 are generally accepted as sufficient^20^ , thus as shown in Fig 2 (lower panels) for all cell lines increasing the number of normalization genes from 2 to 3 is beneficial but unnecessary.

**geNorm analysis:**
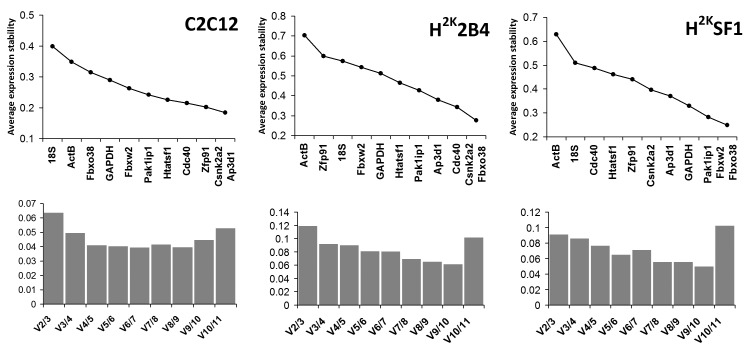
Upper panel: Genes ranked by their calculated average expression stabilities (stability increases from left to right) in C2C12, 2B4 and SF1 (left to right, respectively). Lower panel: optimal number of control genes for normalization for C2C12, 2B4 and SF1 (left to right, respectively). Charts compare pairwise variation V of normalization factors as number of factors used increases (n/n+1). V2/3 thus compares 2 genes with 3, V3/4: 3 genes with 4. Values below 0.15 are assumed to be sufficient, thus in all cases increasing the number of genes used beyond 2 is of limited benefit.

**Normfinder** analysis (Fig 3) confirmed that C2C12 cells exhibit greater overall expression stability than the immortomouse lines, and again **Csnk2a2** and **Ap3d1** scored highly in all lines. This analysis also produced greater consensus on suggested candidates, with **Csnk2a2** universally being the highest scoring single-gene candidate. Furthermore, **18S**, **ActB** and (to a lesser extent) **GAPDH** were again shown to be candidates exhibiting poor stability.

**Normfinder analysis:**
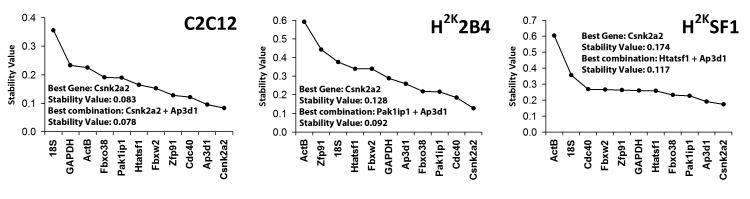
Genes ranked by stability value (summed inter and intra-group stability -Stability increases from left to right) in C2C12, 2B4 and SF1 (left to right, respectively). Suggested candidates and derived stability values for single reference gene or reference gene combination are indicated.

**Bestkeeper** analysis was largely in agreement with the above two methods: while the bestkeeper method generates a substantial amount of data (including individual gene variances and systematic pairwise comparisons between each gene), the most useful output measure for this study is the generation of a global normalization factor derived from the entire dataset (the ‘bestkeeper’) and the Pearson correlation coefficients between each gene and this factor: simply, the degree to which individual genes agree with this calculated global value. As shown in Fig 4, **Csnk2a2** and **Ap3d1** show high degrees of correlation in all three cell lines, and **18S**, **ActB** and **GAPDH** perform poorly. Of particular note is the markedly poor scoring of **18S** in SF1 and C2C12 cells, highlighting the risk of using ribosomal RNA as a standard for messenger RNA. Examination of the raw expression data reveals that while levels of 18S RNA are comparatively consistent -at most a 2-fold variation between highest and lowest signal- (as might be expected given initial amounts of RNA for cDNA synthesis are based on total RNA, which is primarily ribosomal), these levels correlate very poorly with expression data from every messenger RNA measured. Conversely, the relative levels of mRNAs in the dataset correlate strongly with each other: ribosomal RNA and messenger RNA do not strongly correlate, and should not be considered to be coupled.

**Bestkeeper analysis:**
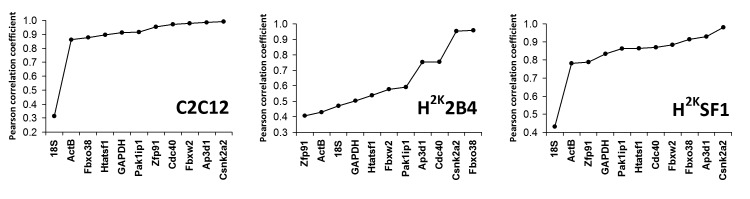
Genes ranked by increasing Pearson correlation coefficient (r) with the bestkeeper values (a series derived from the geometric average of all candidate gene series). Correlation increases from left to right.

Taking these data as a whole, it is tempting (albeit mathematically unsound) to simply sum the data from these three analyses to obtain a ‘consensus’ panel of suitable reference genes. However as noted above each analysis method employs differing approaches, and the confidence of the respective results returned will vary depending on the innate variability of the dataset presented. For example, a gene exhibiting very little variation from the dataset average across all timepoints and replicates would score highly via Normfinder, but (assuming this stability was unique to a single gene in the dataset) would score poorly via geNorm due to lack of a suitable pairwise match. Nevertheless, if a given gene consistently scores highly in all three analyses, and indeed scores highly in all three cell lines, it is likely to be a strong candidate.

A more practical test of the suitability of a given suggested reference gene therefore is an empirical one: at the most basic, one can simply examine the raw expression data for the genes in question. While these data are (by definition) not normalized and thus highly noisy, a differentiation-related trend observable even at this early stage would certainly call into question the suitability of a given genes as a normalization candidate, particularly if such a trend was observed in multiple cell lines. As shown in Fig 5, in all cell lines (albeit to a lower extent in C2C12, where the variation of the entire dataset is lower) **ActB** exhibits a differentiation-correlated decline in expression even when analysed as raw data, while in contrast the expression of the high-scoring candidate **Csnk2a2** does not show any significant trend.

**Csnk2a2 vs ActB : raw expression data:**
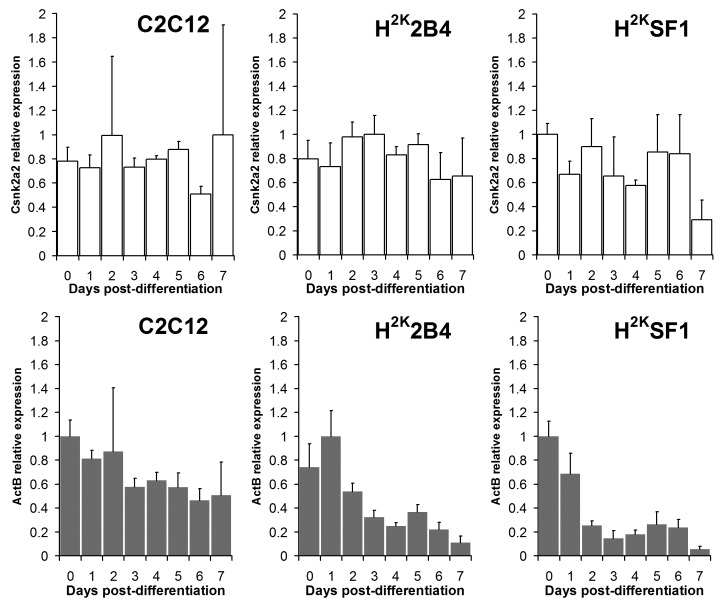
Cq values from Csnk2a2 and ActB expression transformed to a linear range, plotted as means (+standard deviations) of experimental replicates from each timepoint (indicated). Csnk2a2 expression (white bars, top) shows no clear trend. ActB expression (grey bars, bottom) declines progressively throughout differentiation.

To assess the efficacy of the candidate reference genes we measured expression of several genes known to vary over the course of myogenic differentiation, and examined the data without normalization, or normalized using either a strong (**Csnk2a2**) or weak candidate (**ActB**). Note that it is advisable to use the geometric average of several appropriate reference genes for normalization: we have used single gene normalizations here to contrast strong and weak candidates -though the authors note that in this instance, combining **Csnk2a2** with a second high scoring candidate (**Ap3d1**) gave minimal improvement from **Csnk2a2** alone (supplementary figure 3).

As shown in figures 6 and 7 and supplementary figures 1 and 2, both the high-scoring **Csnk2a2 **and the low-scoring **ActB **were often similarly effective in lowering errors within a given timepoint (i.e. normalizing data between replicates). This is to be expected: normalizing expression to *any *mRNA will tend to stabilise apparent signal within a given timepoint regardless of the temporal relationship between mRNAs over the course of differentiation. However, as predicted by the raw analysis above, normalizing using **ActB **produced significantly higher fold changes: consistently lowering apparent early expression signal while concomitantly raising late expression. This is demonstrated particularly effectively via dystrophin expression (Fig 6): the late-differentiation marker dystrophin is an exceptionally large gene (taking ca. 16 hours to fully transcribe) and is spliced co-transcriptionally^22^ . Using a primer set designed to early sequence (the primers used for this study span exons 1-3) will thus detect both completed transcripts and those mid-synthesis. Correspondingly, as dystrophin transcripts in the dystrophic cell line (SF1) will be subject to nonsense-mediated decay, expression in these cells should effectively reflect mid-synthesis transcripts only. Using **Csnk2a2 **to normalize expression data, both C2C12 and H^2K^2B4 cells show an approximately 5-fold increase in dystrophin expression, compared with only 3-fold in SF1 cells, as might reasonably be expected. Furthermore, dystrophin expression appears to plateau by day 4-6 (earlier in 2B4/SF1, later in C2C12). Employing **ActB **as a reference instead results in a less-plausible 10, 25 and 14-fold increase in dystrophin expression in C2C12, 2B4 and SF1 respectively, including a dramatic but physiologically unlikely 2-fold spike in expression between day 6 and 7 in 2B4 cells. Similarly Mef2C, a gene activated early in differentiation^23^ (Fig 7) normalized using **Csnk2a2 **peaks sharply at an approximately 10 to 12-fold increase in all cell lines (again peaking roughly 2 days later in C2C12), whereas when normalized using **ActB**, expression increases 20 to 50 fold with no clear consensus between cell lines. Similar results were observed for myogenin and MyoD (supplementary figures 1 and 2), though the authors note that under these condition MyoD expression appears to change very little in C2C12 cells: indeed myogenic changes in expression as a whole appear to occur earlier, and more dramatically, in the H^2K ^lines than in C2C12 cells. This likely reflects the more synchronised commitment to differentiation effected by the temperature shift and interferon withdrawal protocol used with immortomouse cells (resulting in a more homogenous cell population).

**Dystrophin expression:**
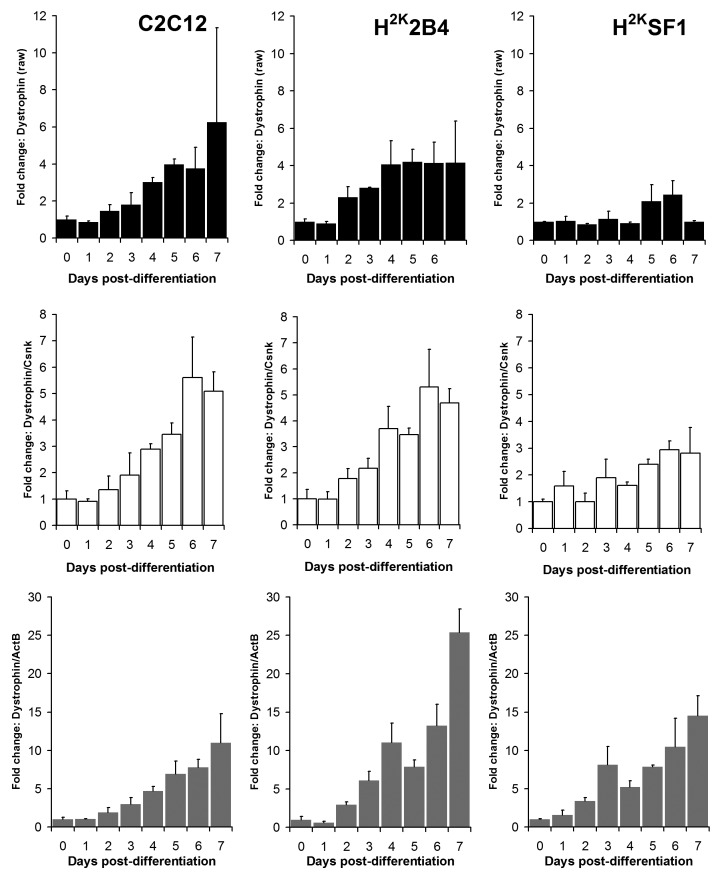
Relative dystrophin expression levels in C2C12 (left) 2B4 (centre) and SF1 (right) cell lines. Data expressed as means (+standard deviations) of linearised values for raw data (black bars), or raw data normalized using Csnk2a2 (white bars) or ActB (grey bars). All data presented as fold change from undifferentiated (day 0) expression level.

**Mef2C expression:**
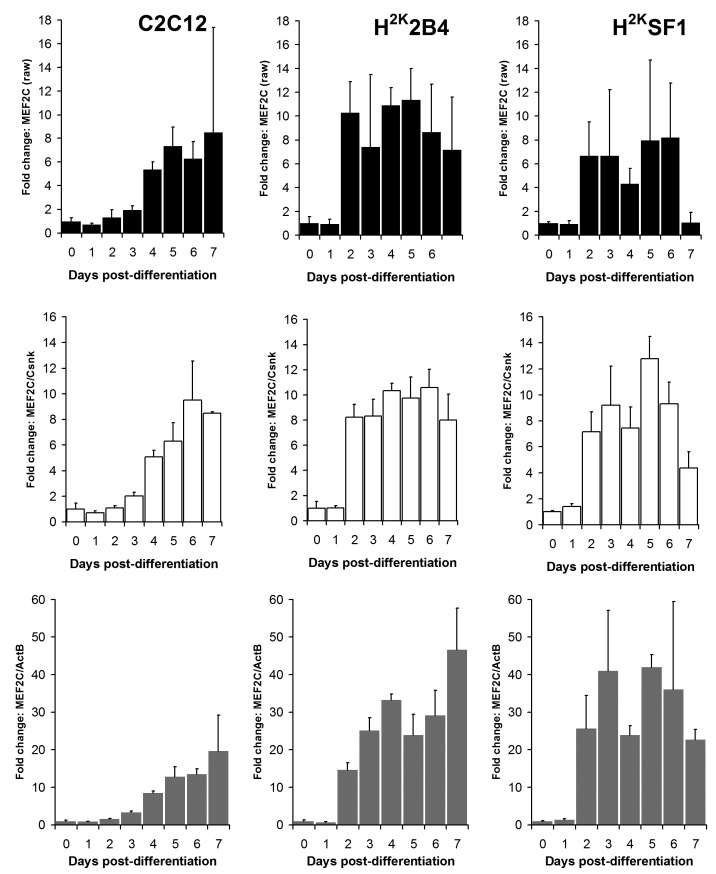
Relative Mef2C expression levels in C2C12 (left) 2B4 (centre) and SF1 (right) cell lines. Data expressed as means (+standard deviations) of linearised values for raw data (black bars), or raw data normalized using Csnk2a2 (white bars) or ActB (grey bars). All data presented as fold change from undifferentiated (day 0) expression level.

## Conclusion

The data presented here suggest **Csnk2a2** is a strong candidate for normalizing expression data throughout myogenesis in cell culture, in both healthy and dystrophic models. **Ap3d1** also scores highly and would thus serve as a suitable additional reference gene, however for this study we found that **Csnk2a2** alone was sufficient. A number of genes appeared to show cell-line specific behaviour: **Fbxo38** and **Pak1ip1** both score highly in immortomouse lines (2B4, SF1) yet poorly in C2C12, while **Zfp91** exhibits the reverse. Similarly, some genes appear to be sensitive to disease-state: **Cdc40** scores highly in healthy cells (2B4, C2C12) and poorly in the dystrophic cell line SF1. Finally, in all cases **18S** RNA demonstrated low correlation with mRNA levels, suggesting that (in contrast to other cell culture studies) for normalizing expression during *in vitro* myogenesis, use of this ribosomal subunit as a reference is not recommended. Within the messenger RNAs, beta actin (**ActB**) scored consistently poorly, and indeed demonstrated a strong differentiation-correlated decrease in expression. The third commonly-cited reference gene **GAPDH** scored marginally higher, though still comparatively poorly: analysis of the raw data (supplementary figure 4) indicates **GAPDH** expression increases markedly over the first 4 days of differentiation.

It should be noted that along with **18S** RNA (which comprises a large fraction of total RNA) both **ActB** and **GAPDH** mRNAs are present at substantially higher levels than the other reference genes investigated: indeed in the case of **GAPDH** by two orders of magnitude (supplementary table 1). While expression levels of the myogenic genes used in this study cover a substantial range (with myogenin and MyoD present at levels over 100 times greater than dystrophin) and vary markedly throughout myogenic differentiation, relative levels of these genes are more closely matched by **Csnk2a2** and **Ap3d1** than by **GAPDH** or **18S** (supplementary figure 5). This is advantageous, as abundant transcripts (**GAPDH**, **ActB**) are readily detected even in samples prepared from poor quality RNA, where genes of interest may no longer be detectable. Use of reference genes sharing a similar expression level to genes of interest affords more confidence to any detected signals, and will more readily highlight any deficiencies in sample preparation/handling.

Taken together, these data demonstrate the unreliability of **18S**, **ActB **and **GAPDH **as universal reference genes and illustrate the importance of determining optimal reference genes for any given set of experimental conditions. We show that use of multiple analysis methods with a relatively large panel of candidates allows the identification of appropriate reference genes even in highly dynamic biological scenarios, and that **Csnk2a2 **and **Ap3d1 **are suitable for normalizing gene expression during *in vitro *myogenesis in both healthy and dystrophic cells.

## Figures and Tables

**MyoD expression:**
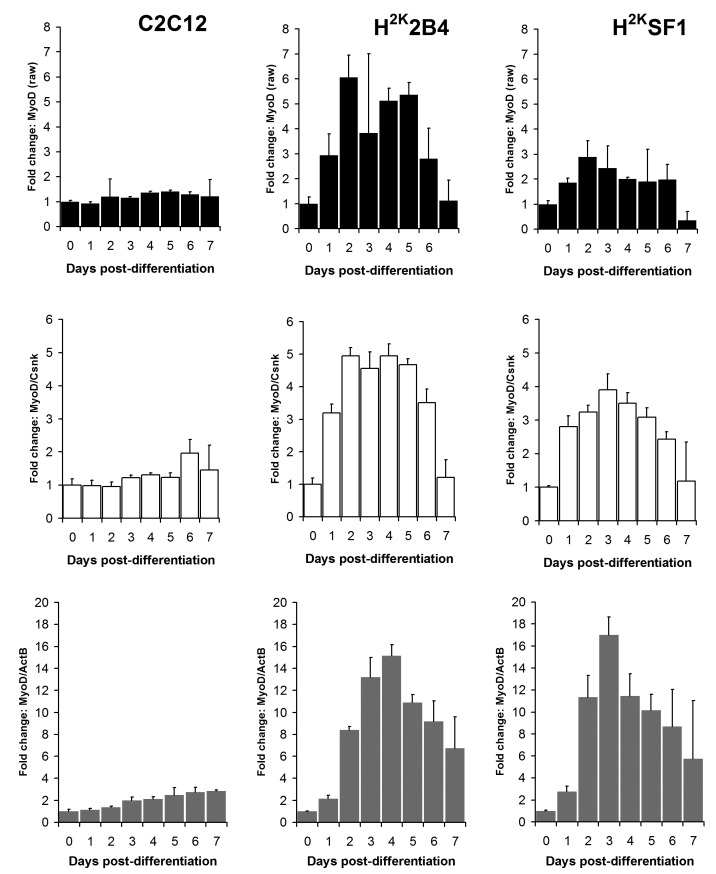
Relative MyoD expression levels in C2C12 (left) 2B4 (centre) and SF1 (right) cell lines. Data expressed as means (+standard deviations) of linearised values for raw data (black bars), or raw data normalized using Csnk2a2 (white bars) or ActB (grey bars). All data presented as fold change from undifferentiated (day 0) expression level.

**Myogenin expression:**
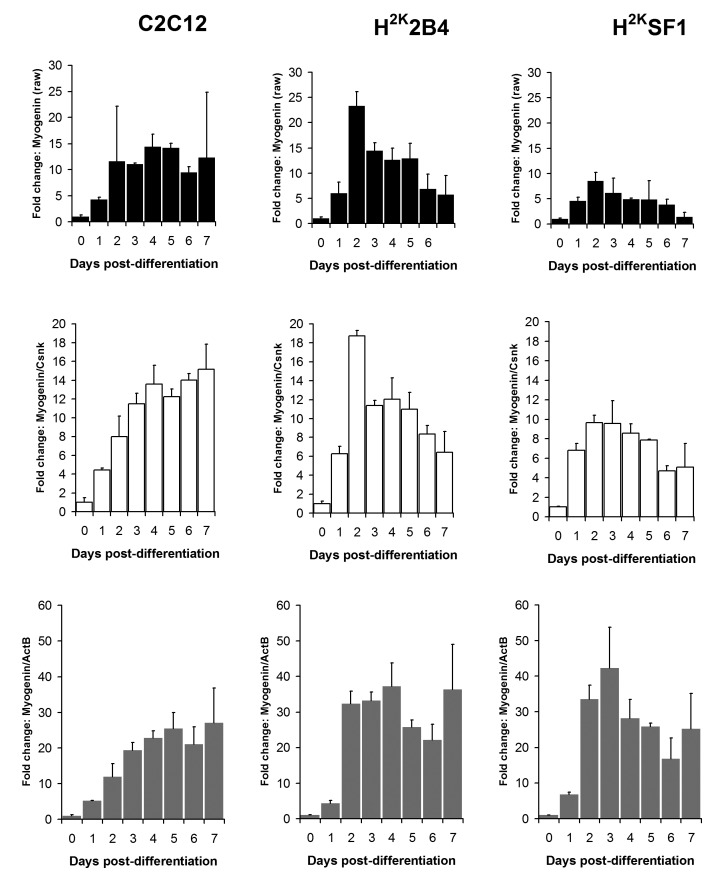
Relative Myogenin expression levels in C2C12 (left) 2B4 (centre) and SF1 (right) cell lines. Data expressed as means (+standard deviations) of linearised values for raw data (black bars), or raw data normalized using Csnk2a2 (white bars) or ActB (grey bars). All data presented as fold change from undifferentiated (day 0) expression level.

**Normalizing with Csnk2a2 alone vs Csnk2a2 and Ap3d1:**
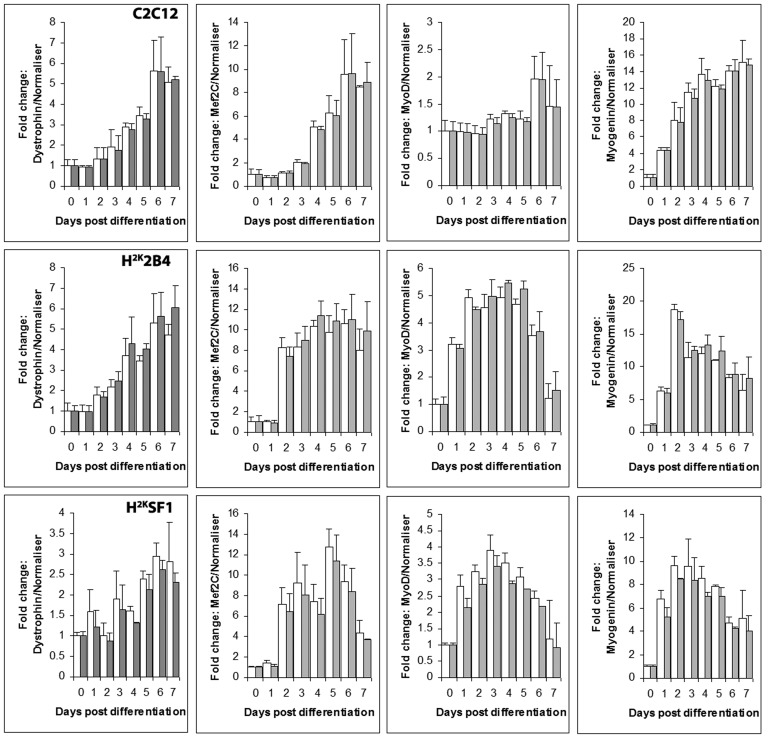
Relative expression levels of dystrophin, MEF2C, MyoD and myogenin (left to right) in C2C12 (top) 2B4 (middle) and SF1 (bottom) cell lines, normalized to Csnk2a2 (white bars) or to the geometric average of Csnk2a2 and Ap3d1 (grey bars). Data expressed as means +standard deviations. All data presented as fold change from undifferentiated (day 0) expression level.

**GAPDH raw expression data:**
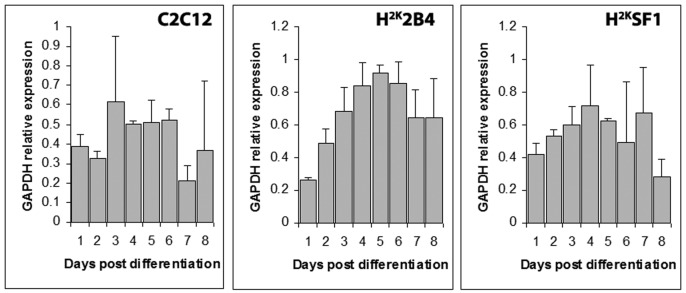
Cq values from GAPDH expression transformed to a linear range, plotted as means (+standard deviations) of experimental replicates from each timepoint (indicated).

**Relative expression ranges:**
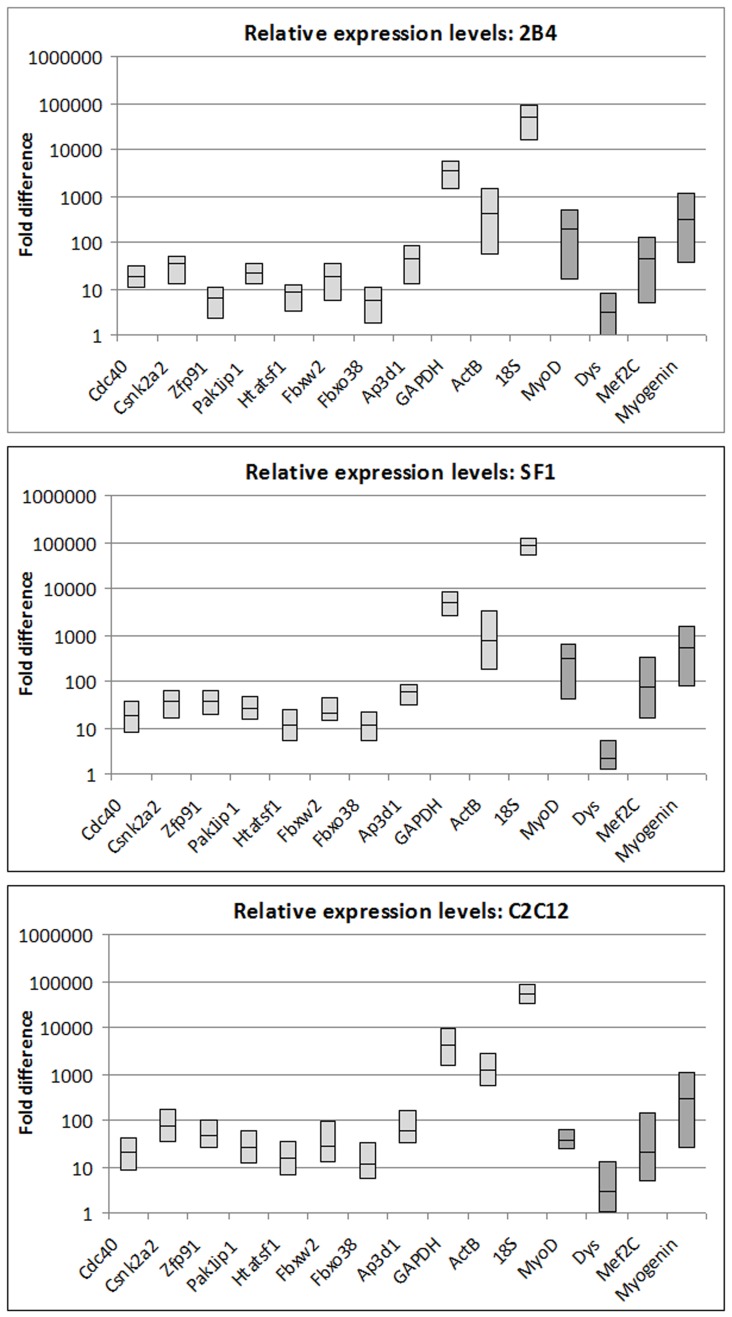
Representative expression levels of the genes used in this study. Midlines indicate mean expression level, highest and lowest levels detected for each gene indicated by bounding box. Expression is calculated as fold change relative to the lowest detected signal (dystrophin), and is displayed on a log(10) scale. Reference genes are displayed in light grey, myogenic genes in dark grey. Top panel: 2B4. Middle panel: SF1. Lower panel: C2C12.

## Appendix 1

### Supplementary Data

**Mean Cq values:** Arithmetic means of Cq values from the eleven reference genes and four myogenic genes used in this study, in the three cell lines investigated. All Cq values were below 30.

	H2K2B4	H2KSF1	C2C12
Cdc40	24.4	24.45	24.22
Csnk2a2	23.45	23.25	22.38
Zfp91	25.93	23.34	22.98
Pak1ip1	24.05	23.82	23.86
Htatsf1	25.44	25.07	24.58
Fbxw2	24.38	24.16	23.71
Fbxo38	26.05	25.06	25.09
Ap3d1	23.06	22.6	22.63
GAPDH	16.8	16.28	16.59
ActB	19.85	18.94	18.3
18S	13.01	12.18	12.86
MyoD	20.94	20.29	23.3
Dystrophin	26.87	27.5	27.04
Mef2C	23.04	22.37	24.14
Myogenin	20.23	19.53	20.34
